# Direct Effects of Glyphosate on *In Vitro* T Helper Cell Differentiation and Cytokine Production

**DOI:** 10.3389/fimmu.2022.854837

**Published:** 2022-03-10

**Authors:** Ambra Maddalon, Martina Iulini, Valentina Galbiati, Claudio Colosio, Stefan Mandić-Rajčević, Emanuela Corsini

**Affiliations:** ^1^ Laboratory of Toxicology, Department of Pharmacological and Biomolecular Sciences, Università degli Studi di Milano, Milan, Italy; ^2^ Occupational Health Unit, International Centre for Rural Health, ASST Santi Paolo e Carlo, Department of Health Sciences, Università degli Studi di Milano, Milan, Italy

**Keywords:** glyphosate, immunotoxicity, T helper cells, estrogenic effect, miR-500a

## Abstract

Glyphosate (G) is the active ingredient of the most used herbicides worldwide. Its use is currently very debated, as several studies indicating its hazard and toxicity are emerging. Among them, there is evidence of adverse effects on the immune system. The aim of this work was to investigate if G could directly affect immune cells. Peripheral blood mononuclear cells (PBMC) obtained from healthy donors were used as experimental model. PBMC were expose to G and stimulated with PMA/ionomycin, T helper (Th) cell differentiation and cytokine production were assessed by flow cytometry and enzyme-linked immunosorbent assay, respectively. A reduction of Th1/Th2 ratio, mainly due to a decrease in Th1 cells, was observed following G exposure. Results show an enhancement of IL-4 and IL-17A production, and a reduction of IFN-γ. Based on literature evidence that suggest G being an endocrine disruptor, we investigated the role of nuclear estrogen receptors (ER). ERα/ERβ inhibition by ICI 182,780 abolished the effects of G on IFN-γ and IL-4 release, suggesting a role of ER in the observed effects. To further characterize the mechanism of action of G, miRNAs, both in exosome and intracellular, were investigated. A statistically significant increase in miR-500a-5p was observed following G treatment. The blockage of miR-500a-5p, using a specific antagomir, prevented G-induced reduction of IFN-γ production. Finally a relationship between miR-500a-5p up-regulation and ER was observed. Overall, these results suggest that G can directly act on T cells, altering T cell differentiation and cytokines production.

## Introduction

Glyphosate (N-(phosphonomethyl) glycine; G) is the active ingredient of the most used broad-spectrum herbicide worldwide (1, 2). It was commercialized starting from 1974 under the name of Roundup^®^, together with adjuvants, like polyoxyethylene tallowamine, that, although considered inert compounds, can increase G toxicity (3).

Its success further increased following the introduction of genetically modified G-resistant crops (4). The herbicidal activity is based on the competitive inhibition of the shikimate pathway, which leads to the blockage of the synthesis of aromatic amino acids in plants (5). Since animals do not have this pathway, G has always been considered safe for humans by regulatory agencies (6, 7).

However, evidence of G toxicity and its alleged threats to the ecosystem and human health are emerging (8, 9). Humans can be exposed to G through different routes; mainly orally, but also through dermal and inhalation exposures (10, 11). Although the majority of studies addressing G toxicity used high doses, there are also studies using environmental relevant doses showing noxious effects in both animals and humans (12–16).

As for general toxicity, there is also evidence of adverse effects against the immune system. Indeed, the latter can be the target of several pesticides (17–19). G-induced immunotoxicity could be the result of a direct action of the herbicide, or can be due to an indirect mechanism, secondary to endocrine, nervous system, or microbiota alterations (20–22). Regarding mammals, evidence indicates the ability of G and G-based herbicides to alter immune responses and to induce inflammation (22). More specifically, intranasal injection of G-rich air samples in mice enhanced eosinophils, neutrophils, and mast cells degranulation in lungs, suggesting an asthma-like pathology (23). In this study, an activation of T helper (Th) 2 response, as reported by the higher production of Th2 type cytokines in lungs, namely IL-4, IL-5, IL-10, IL-13 and IL-33, was observed. Roundup^®^ was shown to induce pro-inflammatory cytokines (i.e., IL-1β and TNF-α) in liver of rats exposed *via* feeding at the doses of 100 and 250 mg/kg bw/day (24). Similarly, the exposure *via* gavage was able to increase the mRNA levels of IL-1β, IL-6, TNF-α, MAPK3, NF-κB, and caspase-3 in the jejunum of rats exposed to the doses of 50 and 500 mg/kg bw/day (25). Authors hypothesized a role of microbiota in G-induced immunotoxicity, as a significant decrease in the relative abundance of Firmicutes and Lactobacillus, while an enrichment in several potentially pathogenic bacteria were observed (25). The same result was obtained by Qiu etal. (26), which treating piglets with Roundup^®^ in diet, observed increased mRNAs of IL-6 and NF-κB in the jejunum following the exposure to 10, 20 and 40 mg/kg bw/day.

Beside animal studies, there are also few epidemiological studies supporting G-based herbicides affecting human immune system. The Agricultural Health Study demonstrated an association between G occupational exposure and current rhinitis and increased rhinitis episodes (27, 28). This study revealed also an association between allergic and non-allergic wheezes among G sprayers (29).

As microbiota is known to influence the immune system and considering that the shikimate pathway is also found in some microorganisms, the interaction of G with commensal microorganisms represents a plausible explanation of G-induced immunotoxicity. Indeed, recently G-based herbicide ability to inhibit the shikimate pathway in the microbiome was demonstrated (30). Furthermore, Roundup LP Plus^®^ was demonstrated to alter the metabolism of *E. coli*, that in turn impacted on the inflammatory immune response, inducing a higher production of TNF-α (31). In addition, associations between G and immune-endocrine disturbances have been described, which also represent a plausible mechanism underlaying G immunotoxicity (reviewed in 22).

The aim of this study was, therefore, to investigate if G could directly affect T helper (Th) cell differentiation and functions. The study focused on Th cells as they have been identified in mice as a possible target of G (23). Investigations were also conducted to get insights on the underlying mechanism of action focusing on the role of the estrogen receptor (ER) and miRNAs in G-induced immunotoxicity. Results obtained demonstrated that G can directly act on T cells, altering T cell differentiation and cytokines production, with a role of estrogen receptor and miR-500-5p in the observed effects.

## Materials and methods

### Chemicals

G (CAS #1071-83-6, purity ≥ 99%) was purchased from Sigma-Aldrich (St. Louis, MO, USA). It was dissolved in Dulbecco’s Phosphate Buffered Saline. Phorbol 12-myristate 13-acetate (PMA – CAS #16561-29-8, purity ≥ 99%) and ionomycin from *Streptomyces conglobatus* (CAS #56092-81-0, purity ≥ 98%) were purchased from Sigma-Aldrich and used to stimulate immune cells, as well as phytohaemagglutinin (PHA, CAS #9008-97-3). 17β-estradiol (E2 – CAS #50-28-2, purity 98%) was used as reference compound for ER activity, and it was obtained from Sigma-Aldrich. ICI 182,780 (CAS # 129453-61-8, purity ≥ 99%) was used as ER antagonist and it was obtained from Tocris Bioscience (Bristol, United Kingdom). All the substances, with the exception of G, were dissolved in dimethyl sulfoxide (DMSO, CAS # 67-68-5, purity ≥ 99.5%), with a final DMSO concentration in culture medium ≤ 0.2%. Cell culture medium and all supplements were also purchased from Sigma-Aldrich.

### Cells

Peripheral blood mononuclear cells (PBMC) were obtained by Ficoll gradient centrifugation from anonymous buffy coats of 5 male healthy donors, purchased from the Niguarda Hospital in Milan (Italy). After centrifugation, PBMC layers were removed and washed 5 times with Dulbecco’s Phosphate Buffered Saline. Isolated cells were diluted to 10^6^ cells/mL in RPMI 1640 with phenol red containing 2 mM L-glutamine, 0.1 mg/mL streptomycin, 100 IU/mL penicillin, gentamycin 10 µg/mL, 50 µM 2-mercaptoethanol, supplemented with 10% heated-inactivated fetal bovine serum (culture media) and cultured at 37°C in 5% CO_2_ incubator. Experiments with E2 and ICI 182,780 were performed using RPMI 1640 without phenol red containing 2 mM L-glutamine, 0.1 mg/mL streptomycin, 100 IU/mL penicillin, gentamycin 10 µg/mL, 50 µM 2-mercaptoethanol, supplemented with 5% heat-inactivated dialyzed fetal bovine serum. Preliminary experiments were conducted to identify non-cytotoxic concentrations (cell viability > 90%). Cytotoxicity was assessed by LDH Cytotoxicity Detection Kit (Takara Bio USA, Inc.) (data not shown).

### Th Cells Analysis

Phenotyping of Th cells was conducted using the Human Th1/Th2/Th17 Phenotyping Kit (BD Pharmingen, Inc.). PBMC (10^6^ cells/mL) were incubated overnight (o.n.) and then treated in the presence or absence of G at the concentrations of 0.01, 0.1, 1 and 10 µg/mL. After 1 hour, cells were stimulated with PMA (50 ng/mL), ionomycin (1 µg/mL) and GolgiStop™ Protein Transport Inhibitor for 5 hours, following manufacturer’s instructions. After treatment, cells were centrifuged at 1300 rpm for 5 minutes and stained with anti-human CD4 PerCP-Cy5.5 conjugated, anti-IL-17A-PE, anti-IL-4-APC and anti-IFN-γ-FITC following manufacturer’s instructions. Cells were analyzed using NovoCyte 3000 flow cytometer, and data were quantified using NovoExpress software (Acea Biosciences, Inc). Results are expressed as fold-change of G-treated versus control cells.

### Cytokine Production

PBMC (10^6^ cells/mL) were incubated o.n. and then treated with G at the concentrations of 0.01, 0.1, 1 and 10 µg/mL. After 1 hour, cells were stimulated with PMA (10 ng/mL) and ionomycin (100 ng/mL) or PHA (1.2 μg/mL) for 72 hours. Following treatment, cells were centrifuged at 1300 rpm for 5 minutes and supernatants collected for cytokine measurement and stored at -20°C until measurement. Cytokine production was assessed in cell-free supernatants by specific sandwich enzyme-linked immunosorbent assays (ELISA) commercially available. ELISA for IL-4 and IFN-γ were purchased from ImmunoTools (Friesoythe, Germany), while IL-17A ELISA from BioLegend (San Diego, CA, USA). Antibodies dilutions were performed according to manufacturer’s instructions. Limits of detection were 2.3 pg/mL for IL-4, 24 pg/mL for IFN-γ, and 3.9 pg/mL for IL-17A. Results are expressed as fold-change of released cytokines of G-treated versus control cells.

### miRNAs Contained in Exosomes Analysis

PBMC (3 x 10^6^) were incubated o.n. and then treated with G at the concentration of 0.1 µg/mL for 72 hours in culture medium without fetal bovine serum. Following treatment, PBMC were centrifuged at 260*g* at 25°C for 5 minutes and supernatants used for exosome extraction. Cell-free supernatants were centrifuged at 20’000*g* at 4°C for 30 minutes, and supernatants centrifuged again at 100’000*g* at 4°C for 90 minutes. The pellet obtained represented the exosomes released by PBMC. Total RNA was extracted using RNeasy Plus Mini Kit (Qiagen, Valencia, CA, USA) following manufacturer’s instructions. Retro-transcription was performed according to manufacturer’s instructions (miScript II RT Kit, Qiagen). Before Real-Time PCR, the selected miRNAs were pre-amplified using the miScript PreAMP PCR kit (Qiagen). For PCR analysis miScript SYBR^®^ Green PCR Kit (Qiagen) was used. All the primers were purchased from Qiagen and used according to manufacturer’s instructions. The quantification of the miRNAs was performed by the 2^−ΔΔCT^ method. The fold-changes of 7 miRNAs (hsa_miR-10b, hsa_miR-27a, hsa_miR-100, hsa_miR-136, hsa-miR-424, hsa_miR-500a, hsa_let-7f) were analyzed, and miRNA hsa-RNU6-2 was used for normalization.

### Intracellular miR-500a Analysis

Following the above-mentioned treatment and centrifugation at 260*g* at 25°C for 5 minutes, total RNA was extracted from the pellet, using RNeasy Plus Mini Kit. RNA was retro-transcribed and the fold-change of hsa_miR-500a was analyzed using miScript SYBR^®^ Green PCR Kit. The quantification was performed by the 2^−ΔΔCT^ method, using miRNA hsa-RNU6-2 for normalization.

### miRNA Silencing

Hsa_miR-500a-5p was silenced through the use of miRCURY LNA™ miRNA inhibitor (Qiagen). 400’000 cells, after o.n. incubation, were silenced with 25 pmol of miRNA inhibitor and 3 µl of HiPerFect Transfection Reagent (Qiagen) for 24 hours. Then, the medium was replaced, and cells treated in the presence or absence of G (0.1 µg/mL) for 1 hour, and then stimulated with PMA and ionomycin for 72 hours. Following treatment, cells were centrifuged at 1300 rpm for 5 minutes and supernatants collected for IFN-γ measurement.

### Data Analysis

With exception of PHA-induced cytokine release (n=3), all experiments were conducted using 5 donors. Data are expressed as mean ± standard error (SEM). Statistical analysis was performed using GraphPad Prism version 9.1.1 (GraphPad Software, La Jolla, CA, USA). Data were analyzed by One-way analysis of variance (ANOVA) followed by Dunnett’s multiple comparison test or Tukey’s multiple comparison test or by paired Student’s t test. Differences were considered significant at *p* ≤ 0.05.

## Results

### Effects of G on Th Differentiation

**Figure 1:**
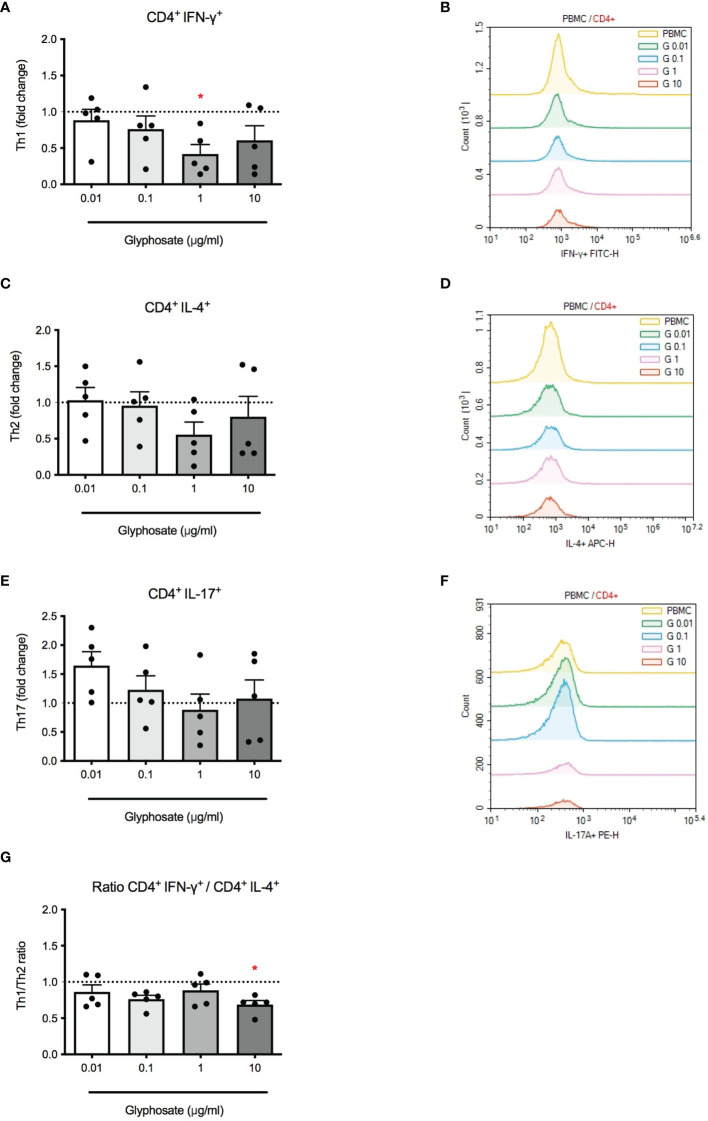
Effect of G on PMA plus ionomycin-induced CD4^+^ cell differentiation. PBMC (10^6^/mL) were treated for 1 h with increasing concentrations of G (0.01, 0.1, 1 and 10 µg/mL), and then stimulated with PMA, ionomycin and GolgiStop™ for 5 h, as described in the Materials and Methods section. **(A)** CD4^+^ IFN-γ^+^ cells. **(C)** ^+^ IL-4^+^ cells. **(E)** CD4^+^ IL-17A^+^ cells. **(G)** CD4^+^ IFN-γ^+^/CD4^+^ IL-4^+^ cell ratio. Results are expressed as fold-change of the number of positive cells in G treated cells compared to control cells. The dotted line reported is set at 1.0 (control). Each value represents the mean ± SEM, *n* = 5 donors. Each dot represents the value of the single individual. Statistical analysis was performed with Dunnett’s multiple comparison test, with *p < 0.05 vs control cells. Flow cytometric analysis of representative histogram overlay were reported in **(B, D, F)**.

Epidemiological studies (27, 29) and mice study (23) suggest that G may be associated with increased risk of allergic reactions with increased Th2 responses. To assess if G was able to directly affect immune cells, its effects on Th differentiation were evaluated *in vitro*. PBMC were treated with G alone at increasing concentrations for 1 hour and stimulated with PMA and ionomycin for subsequent 5 hours. IFN-γ, IL-4 and IL-17A positive cells were analyzed by flow cytometry (**Figure 1**). Gating strategies for flow cytometry panels are reported in **Supplementary Material** (**Supplementary Figure 1**). A reduction of IFN-γ positive cells was observed following G treatment that reached statistically significance at 1 µg/mL (**Figure 1A**). No significant changes were observed in IL-4 positive cells (**Figure 1B**). When analyzing the ratio between IFN-γ and IL-4 positive cells (**Figure 1C**), a decrease was observed, reaching the statistical significance at the concentration of 10 µg/mL. No statistically significant effects were observed in IL-17A positive cells (**Figure 1D**). Overall, data indicate that G can directly affect Th cell differentiation, resulting in an imbalance of Th1/Th2 subpopulations, supporting an increase in Th2 responses.

### Effects of G on Cytokine Production

**Figure 2:**
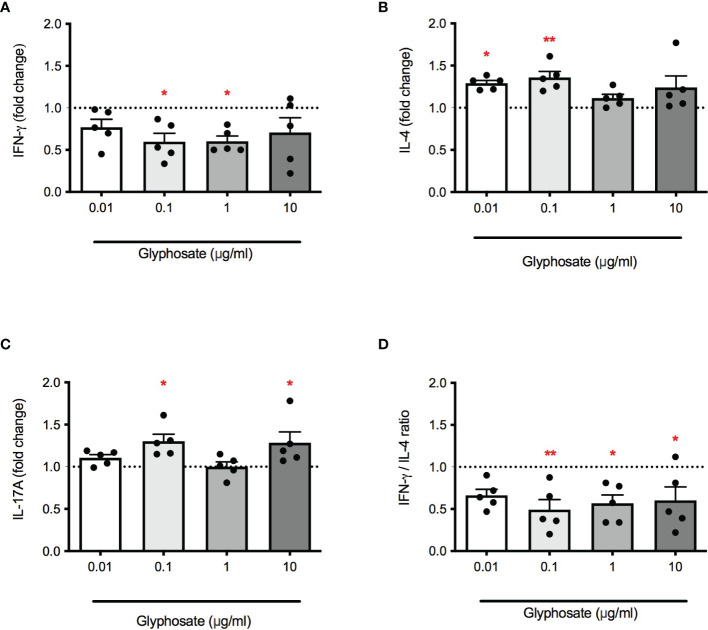
Effect of G on PMA plus ionomycin-induced IFN-γ, IL-4 and IL-17A release. PBMC (10^6^/mL) were treated for 1 h with increasing concentrations of G (0.01, 0.1, 1 and 10 µg/mL), and then stimulated with PMA and ionomycin for 72 h. Cytokines were measured by ELISA in cell-free supernatants. **(A)** IFN-γ release. **(B)** IL-4 release. **(C)** IL-17A release. **(D)** IFN-γ/IL-4 ratio. Results are expressed as fold-change of the released cytokines in G treated cells compared to control cells. The dotted line reported is set at 1.0 (control). Each value represents the mean ± SEM, *n* = 5 donors. Each dot represents the value of the single individual. Statistical analysis was performed with Dunnett’s multiple comparison test, with *p < 0.05 and **p < 0.01 vs control cells.

Next, the effect of G on cytokine release, namely IFN-γ, IL-4, IL-17A, in response to PMA + ionomycin or PHA was investigated. PBMC were treated with increasing concentrations of G alone for 1 hour and then stimulated with PMA and ionomycin or PHA for 72 hours. Results were overall consistent with data shown in **Figure 1**. G reduced IFN-γ release (**Figure 2A**) which resulted statistically significant at 0.1 and 1 µg/mL. The release of IL-4 (**Figure 2B**) was enhanced, although not linearly, reaching statistically significance at the concentrations of 0.01 and 0.1 µg/mL. This imbalance in IFN-γ and IL-4 release resulted in a reduction in the IFN-γ/IL-4 ratio (**Figure 2C**), indicating an action in favor of Th2 cells and against Th1 cells. Regarding IL-17A production (**Figure 2D**), a statistically significant increase was observed at the concentrations of 0.1 and 10 µg/mL, with no clear dose-response. Similar results were also observed using PHA (**Table 1**), a lectin known to bind to T-cell membranes stimulating activation and proliferation (32), confirming the ability of G to unbalance Th1/Th2 responses, favoring Th2.

**Table 1:** Effect of G on PHA-induced cytokine production.

Treatment (μg/mL)	IFN-γ	IL-4	IFN-γ/IL-4 ratio
**0.01**	0.51 ± 0.22*	0.76 ± 0.05	0.65 ± 0.24
**0.1**	0.38 ± 0.13**	0.91 ± 0.16	0.38 ± 0.07**
**1.0**	0.58 ± 0.28	0.90 ± 0.23	0.43 ± 0.15**
**10.0**	0.67 ± 0.18	0.89 ± 0.17	0.58 ± 0.16*

PBMC (10^6^/mL) were treated for 1 h with increasing concentrations of G (0.01, 0.1, 1 and 10 µg/mL), and then stimulated with PHA (1.2 µg/mL) for 72 h. Cytokines were measured by ELISA in cell-free supernatants. Results are expressed as fold changes vs cells treated with PHA alone. Each value represents the mean ± SEM, n = 3 donors. Statistical analysis was performed with Dunnett’s multiple comparison test, with *p < 0.05 and **p < 0.01 vs control cells.

Our findings demonstrated a non-monotonic response of G on some endpoints of Th differentiation and cytokine production. For instance, lower concentrations can exert higher effects, with a non-linear trend.

Based on these results, subsequent experiments were conducted using G at the concentration of 0.1 µg/mL. This concentration is biologically relevant as serum level of 0.1891 μg/mL were detected in Thai women involved in agricultural activities (33).

### Role of ER in G-Induced Effects

**Figure 3:**
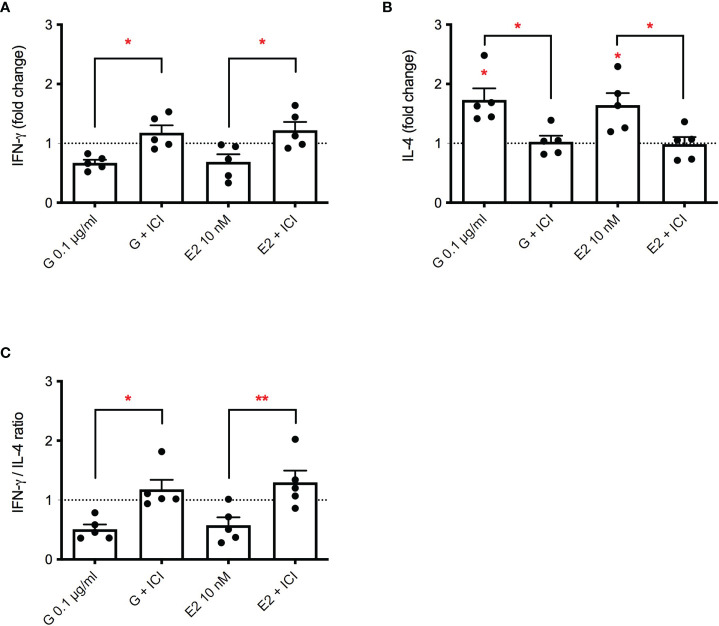
Role of ERα in G-induced cytokine release. PBMC (10^6^/mL) were treated with ICI 182,780 (1 µM) for 15 minutes, and then with G (0.1 µg/mL) or with E2 (10 nM) for 1 h. After that, cells were stimulated with PMA plus ionomycin for 72 h. Cytokines were measured by ELISA in cell-free supernatants. **(A)** IFN-γ release. **(B)** IL-4 release. **(C)** IFN-γ/IL-4 ratio. Results are expressed as fold-change of the cytokine released in G treated cells versus relative control cells (untreated cells for G- or E2-treated cells and cells treated with ICI for G+ICI- or E2+ICI-treated cells). The dotted line reported is set at 1.0 (control). Each column represents the mean ± SEM, *n* = 5 donors. Each dot represents the value of the single donor. Statistical analysis was performed with Tukey’s multiple comparison test, with *p < 0.05 and **p < 0.01 vs control untreated cells or the corresponding not ICI-treated cells.

Even if debated, some literature data suggest that G may act through the estrogen receptor (ER) (34). To investigate a possible role of ER in G-induced immunotoxicity the ER inhibitor ICI 182,780 was used (35). E2 was used as positive control. The release of IFN-γ, IL-4 and their ratio was investigated, following treatment with G at the concentration of 0.1 µg/mL or E2 (10 ng/ml), with a pre-treatment of 15 minutes in the presence or absence of ICI (1 µM). G and E2 similarly reduced the production of PMA-ionomycin induced IFN-γ (**Figure 3A**). ICI pre-treatment combined with G or E2 was able to completely restore IFN-γ production. The same behavior was observed with the release of IL-4 (**Figure 3B**), which was enhanced by both G and E2 and restored by ICI. The decrease in IFN-γ/IL-4 ratio induced by both G and E2, was prevented by ICI, indicating a role of ER in G-induced Th1/Th2 imbalance.

### Role of miR-500a in G-Induced Effects on Lymphocytes

**Figure 4:**
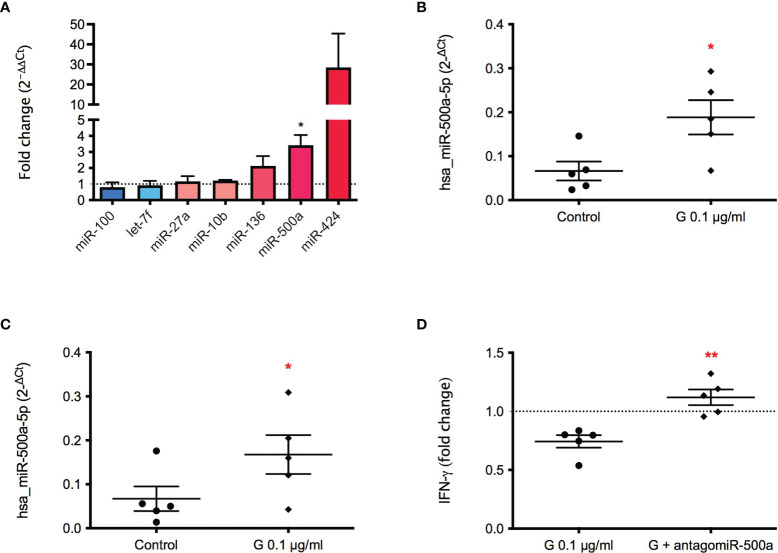
Effect of G on the exosome and intracellular expression of the selected miRNAs. PBMC (10^6^/mL) were treated for 72 h with G (0.1 µg/mL). Exosomes were prepared as described in the Material and Methods section. Total RNA was extracted, retro-transcribed, and target miRNAs were pre-amplified and detected through Real-Time PCR. **(A)** Fold-changes (2^−ΔΔCt^) of the selected miRNAs in exosomes (hsa_miR-10b, hsa_miR-27a, hsa_miR-100, hsa_miR-136, hsa-miR-424, hsa_miR-500a, hsa_let-7f) are reported. **(B)** Fold-change (2^−ΔCt^) of hsa_miR-500a contained in exosomes. **(C)** Fold-change (2^−ΔCt^) of intracellular hsa_miR-500a. hsa-RNU6-2 as negative control. **(D)** Role of miR-500a in IFN-γ production. PBMC (40’000 cells per well) were silenced with the inhibitor of hsa_miR-500a-5p for 24 h, after medium replacement cells were treated with G (0.1 µg/mL) for 1 h and then with PMA and ionomycin for 72 h. IFN-γ was measured by ELISA in cell-free supernatants. In **(B, C)** dots represent the individual values of control cells and rhombuses of G-treated cells for each donor. In **(D)** each dot represents the fold change of IFN-γ release induced by G of each individual, whereas rhombuses represent the fold change of IFN-γ release obtained from antagomir pre-treatment. Each value represents the mean ± SEM, *n* = 5 donors. Statistical analysis was performed with Paired t test, with *p < 0.05 and **p < 0.01 vs control cells (or G-treated cells for **(D)**.

To further investigate the mechanism underlying G immunotoxicity, starting from a miRNA panel conducted on exosomes obtained from plasma samples of farmers occupationally exposed to G-based herbicide (manuscript in preparation), seven de-regulated miRNAs were selected, namely miR-10b, miR-27a, miR-100, miR-136, miR-424, miR-500a, let-7f. PBMC were treated with G (0.1 µg/mL) for 72 hours, and the presence of the selected miRNAs in exosomes investigated (**Figure 4A**). Hsa-miR-100 appeared to be slightly down-regulated following G treatment, whereas the other 6 miRNAs investigated resulted to be up-regulated. Even if miR-424 resulted clearly up regulated, the only statistically significant change was observed for hsa-miR-500a-5p, which expression was over 3 times higher following G exposure (**Figure 4B**). The same over-expression was also observed for the intracellular miRNA (**Figure 4C**), indicating an effect of G both on intracellular and exosome-released miR-500a-5p.

To investigate if hsa-miR-500a-5p was involved in G-induced inhibition of IFN-γ release, miR-500a-5p was silenced by the use of an antagomir. The pre-incubation with hsa-miR-500a-5p inhibitor was able to restore the production of IFN-γ, in a statistically significant manner (**Figure 4D**), indicating a role of miR-500a-5p in the observed effects.

**Figure 5:**
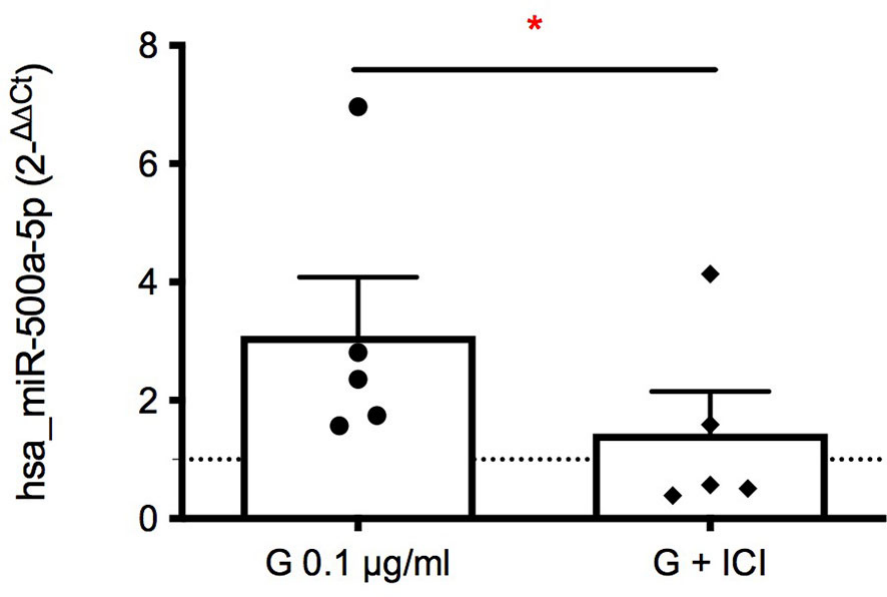
Role of ERα in G-induced intracellular expression of hsa-miR-500a-5p. PBMC (10^6^/mL) were treated with ICI 182,780 (1 µM) for 15 minutes, and subsequently with G (0.1 µg/mL) for 72 h. Total RNA was extracted, retro-transcribed, and hsa-miR-500a-5p detected through Real-Time PCR. Fold-changes (2^−ΔΔCt^) are reported, using hsa-RNU6-2 as negative control. Each dot represents the fold change of hsa-miR-500a-5p expression induced by G over control for each individual, whereas rhombuses represent the fold change of hsa-miR-500a-5p expression obtained from cells treated with ICI 182,780 and G over ICI-treated cells. Each value represents the mean ± SEM, *n* = 5 donors. Statistical analysis was performed with Paired t test, with *p < 0.05 vs G-treated cells.

Therefore, we highlighted an association between hsa-miR-500a-5p and IFN-γ, which release can be also affected by the pre-treatment with ICI 182,780. To correlate these two phenomena, the expression of hsa-miR-500a-5p induced by G was assessed following ER inhibition. The inhibition of ER was able to statistically significantly reduce the expression of hsa-miR-500a-5p in the intracellular compartment (**Figure 5**), indicating the implication of ER in G-induced enhancement of hsa-miR-500a-5p expression. Despite the individual variability of miRNA expression, a reduction of hsa-miR-500a-5p expression, following ER inhibition, can be observed.

## Discussion

The purpose of this study was to investigate the ability of G to directly affect Th lymphocytes. Results demonstrated that G was able to decrease the Th1/Th2 ratio, inhibiting IFN-γ production. Effects could be reversed by the ER antagonist ICI 182,780, supporting a role of endocrine disturbance, and by miR-500a-5p antagomir. Results are in agreement with the study conducted in mice by Kumar et al. (23) that demonstrated that G-rich air samples promoted Th2 type cytokines.

Even if still controversial, the ability of G to activate ER *in vitro* has been demonstrated (34, 36), and confirmations also came from experimental animals, showing ERα activation following herbicide exposure in male and female rats (37, 38). The possible role of ER in G-immunotoxicity is strengthened by the ability of estrogens to influence Th1 and Th2 responses, with a promotion toward Th2 activity (39), and to allergic airway inflammation (40). Estrogens can act on immune cells through both ERα and β (41, 42). ERs are involved in shaping the differentiation of Th cells, affecting transcriptional regulation, with possible consequences in inflammation (43). In this study, the role of ER was demonstrated, by the ability of ICI able to restore IFN-γ and IL-4 modulation, and results further support the endocrine disrupting activity of G (44).

G shown a non-monotonic dose-response relationship in its toxic effects against Th. Many endocrine disruptors, and mainly estrogenic substances, display this characteristic (45), strengthening the hypothesis of G considered as an endocrine disruptor. Some possible explanations to this phenomenon could be the cytotoxicity at high concentrations, the different receptor affinity, or receptor desensitization (45, 46).

To further investigate the mechanism underlying G immunotoxicity, a different expression of miRNAs in exosomes by PBMC following G exposure was investigated. MiRNAs are single-stranded RNAs of about 22 nucleotides, that function as post-transcriptional regulators, through the interference with mRNA processes (47). They can affect both mRNA stability and target mRNA for the subsequent degradation (48). The majority of miRNAs are localized in the intracellular compartment, however several miRNAs in various body fluids have been described (49). Exosomes are miRNAs carriers (50), of great relevance in pharmacology and toxicology (51). Notably, circulating miRNAs have been described as potential biomarkers of autoimmune and inflammatory disease (reviewed by 52). Considering the important role of miRNAs in the regulation of gene expression and their possible application as biomarkers and as cell-cell communication mechanism, we examined the differential expression of several miRNAs in farmers occupationally exposed to G (manuscript in preparation). Results presented in this work suggest miR-500a-5p as a possible target of G in immune cells. Only few studies investigated the effect of G on non-coding RNAs, mainly conducted in mice. Ji et al. (53) associated perinatal G exposure in mice with a differential miRNA expression in the prefrontal cortex (55 miRNAs up-regulated and 19 miRNAs down-regulated), mainly involved in neural development. Also, circular RNAs were affected, with 330 up-regulated and 333 down-regulated (54). Supporting this evidence, more recently, perinatal G exposure in mice was associated with aberrant expression of lncRNAs, highlighting a link with impaired neuronal development (55). Also changes in miRNA profile in female rat liver were assessed following G and G-based herbicide treatment, and interestingly these miRNAs were associated with carcinogenesis, like miR-17 and miR-22 (56). Furthermore, an *in vitro* study performed on carp lymphocytes shown that G-induced lymphocyte apoptosis was mediated by the regulation of miR-203 and PI3K/AKT pathway (57). No studies examined the effect of G on miRNAs in human immune cells, in this regard this study represents the first evidence that G can affect human miRNAs expression linked to the immune system. In particular, a role of miR-500a could be demonstrated, linking miR-500a and lower IFN-γ production. MiR-500a-5p is not a well-characterized miRNA; to date the few available evidence address a role in oxidative stress in breast cancer (58) and in the promotion of breast cancer cell proliferation (59). It has also been described as oncogenic in colorectal cancer (60), as well as responsible for proliferation and metastasis of hepatocarcinoma (61). Based on TargetScan (v7.2; targetscan.org), a useful database to predict miRNA target sites (62), hsa-miR-500a-5p could hypothetically inhibit several mRNAs involved in the differentiation of Th cells towards Th1, preventing it. Among possible targets there are STAT1 (signal transducer and activator of transcription 1), TBX21 (T-box 21; or T-bet), SOCS5 (suppressor of cytokine signaling 5) and ANXA1 (annexin A1), which are all positive regulators of Th1. The up-regulation of miR-500a could be indeed in line with the decrease in Th1 as demonstrated in the current study. Furthermore, we speculate on the existence of a dependance between miR-500a expression and ER, since its inhibition reflected in a lower expression of hsa-miR-500a-5p. Preliminary results indicate indeed a reduced expression of STAT1 following G exposure, which could be reversed by ICI (data not shown), indicating STAT1 as a relevant target to explain G immunotoxicity.

Overall, data obtained indicate that G at biological relevant concentrations can directly affect Th cells, disturbing the balance between Th1 and Th2, through the ER pathway. We also demonstrated a role of miR-500a-5p in G-induced reduced IFN-γ production. Additional studies are required to identify the genes targeted by miR-500a-5p involved in Th1 differentiation and their link with ER.

## Data Availability Statement

The original contributions presented in the study are included in the article/**Supplementary Material**. Further inquiries can be directed to the corresponding author.

## Author Contributions

AM: manuscript writing, reviewing, and editing. MI, VG, CC, SM-R, and EC: manuscript reviewing and editing. All authors contributed to the article and approved the submitted version.

## Funding

Research has been supported by Ministero dell’Istruzione, dell’Università e della Ricerca (PRIN 2017, Project number 2017MLC3NF).

## Conflict of Interest

The authors declare that the research was conducted in the absence of any commercial or financial relationships that could be construed as a potential conflict of interest.

## Publisher’s Note

All claims expressed in this article are solely those of the authors and do not necessarily represent those of their affiliated organizations, or those of the publisher, the editors and the reviewers. Any product that may be evaluated in this article, or claim that may be made by its manufacturer, is not guaranteed or endorsed by the publisher.
