# Supplementary material for: Direct Effects of Glyphosate on In Vitro T Helper Cell Differentiation and Cytokine Production
Source: Front Immunol. 2022 Mar 10;13:854837. doi: 10.3389/fimmu.2022.854837 (PMC8960435; doi:10.3389/fimmu.2022.854837)
Supplement: Supplementary file 1 [file DataSheet_1.docx]

# *Supplementary Material*

**Supplementary Figure 1.**

**Supplementary Figure 1 – Gating strategies for flow cytometry.** Hierarchical gating to identify CD4^+^ IFN-γ^+^, IL-4^+^ and IL-17A^+^ cells. a) Representative dot plot diagram of forward-scatter (FSC) and side-scatter (SSC) light. Threshold level for FSC-H was set to exclude signals from cellular debris. b) Representative dot plot diagram of CD4^+^ cells, indicated by the red gate. c) Representative dot plot diagram of CD4^+^ IFN-γ^+^ and CD4^+^ IL-17A^+^ cells. d) Representative dot plot diagram of CD4^+^ IL-4^+^ and CD4^+^ IL-17A^+^ cells. Plots are for illustration purposes only.
